# Infectivity of Deinbollia mosaic virus, a novel weed-infecting begomovirus in East Africa

**DOI:** 10.1007/s00705-017-3495-x

**Published:** 2017-08-09

**Authors:** Martina Kyallo, Elijah Miinda Ateka, Peter Sseruwagi, José Trinidad Ascencio-Ibáñez, Mildred-Ochwo Ssemakula, Robert Skilton, Joseph Ndunguru

**Affiliations:** 1 Biosciences eastern and central Africa-International Livestock Research Institute (BecA-ILRI) Hub, P.O. Box 30709-00100, Nairobi, Kenya; 2 Department of Horticulture, Jomo Kenyatta University of Agriculture and Technology, P.O. Box 62000-00200, Nairobi, Kenya; 3 Mikocheni Agricultural Research Institute, P.O. Box 6226, Dar es Salaam, Tanzania; 4 Department of Molecular and Structural Biochemistry, North Carolina State University, 132 Polk Hall, Raleigh, NC 27695 USA; 5 School of Agricultural Sciences, Makerere University, P.O. Box 7062, Kampala, Uganda; 6 International Centre of Insect Physiology and Ecology (icipe), P.O. Box 30772-00100, Nairobi, Kenya

## Abstract

Weed-infecting begomoviruses play an important role in the epidemiology of crop diseases because they can potentially infect crops and contribute to the genetic diversity of crop-infecting begomoviruses. Despite the important epidemiological role that weed-infecting begomoviruses play, they remain insufficiently studied in Africa. Recently, we identified Deinbollia mosaic virus (DMV), a distinct begomovirus found naturally infecting the weed host *Deinbollia borbonica* (*Sapindaceae*) in Kenya and Tanzania. In this study, we investigated the capacity of DMV to infect a restricted host range of *Solanaceae* and *Euphorbiaceae* species. Biolistic inoculation of *Nicotiana benthamiana* with concatemeric DNAs resulted in systemic infection associated with yellow mosaic symptoms, while DNA partial dimers caused asymptomatic systemic infection. DMV was not infectious to cassava (*Manihot esculenta* Crantz), suggesting host resistance to the virus. Here, we demonstrate the first experimental infectivity analysis of DMV in *N. benthamiana* and cassava.

## Introduction

The family *Geminiviridae* is divided into nine genera based on genome organization and structure, host range, and insect vector namely *Begomovirus, Mastrevirus, Curtovirus, Becurtovirus, Turncurtovirus, Eragrovirus*, *Grablovirus, Capulavirus*, and *Topocuvirus* [1]. *Begomovirus* is the best described genus and includes emergent pathogens that are widely distributed worldwide and constantly threatening the cultivation of diverse economically important crops [2–4]. Begomoviruses are grouped as monopartite or bipartite depending on the number of genome components they possess. Bipartite begomoviruses have small circular, single-stranded DNA (ssDNA) molecules of about 2.5-2.7 kb known as DNA-A and DNA-B. Genes on DNA-A encode proteins involved in virus replication, gene expression, encapsidation, and vector transmission [5]. DNA-B codes for two proteins involved in virus movement between and within plant cells [6]. Monopartite begomoviruses have a single genomic DNA that is homologous to the DNA-A of the bipartite begomoviruses.

Begomoviruses are transmitted in a persistent and circulative manner to dicotyledonous plant species by the polyphagous whitefly *Bemisia tabaci* (Gennadius) (*Hemiptera*: *Aleyrodidae*). Begomoviruses are often associated with cultivated (crop) and non-cultivated (weed) plants. Weeds may act as natural hosts for many economically important plant viruses and can serve as sources of primary virus inoculum for whitefly transmission [7, 8]. Within the *B. tabaci* complex, the Middle East-Asia Minor 1 cryptic species (MEAM1, formerly B biotype) has been reported to colonize plants belonging to diverse botanical families and may be responsible for the horizontal transfer of begomoviruses between crop and weed plants [9, 10]. Weed-infecting begomoviruses act as progenitors of crop-infecting begomoviruses and contribute to their diversity via genetic recombination [11, 12]. Despite the important role these weed-infecting begomoviruses play in crop diseases, they remain insufficiently studied in Africa.

Deinbollia mosaic virus (DMV) is a phylogenetically conserved bipartite begomovirus that was recently found infecting soapberry (*Deinbollia borbonica*), a non-cultivated plant species in the family *Sapindaceae*, which occurs in the coastal areas of Kenya and Tanzania [13]. Infected plants exhibit typical yellow mosaic symptoms and stunting. The plant host range of DMV has not been determined but may be limited to *D. borbonica*, the only known natural host. The economic importance of DMV has not been established yet. In this paper, we report the infectivity of DMV in plants belonging to the families *Solanaceae* and *Euphorbiaceae*.

## Materials and methods

### Virus source and DNA extraction

Leaf samples were collected from *D. borbonica* seedlings exhibiting yellow mosaic symptoms from a cassava field in northeastern Tanzania (GPS coordinates 05.10219S, 38.47172E; altitude, 202 m; March 2015). The presence of whiteflies on the plants was noted. Total DNA was extracted using the protocol of the ZR Plant/Seed DNA MiniPrep kit (Zymo Research Corp.) according to the manufacturer’s instructions.

### Virus amplification and cloning

Full-length viral genomes were amplified from extracted DNA samples by rolling-circle amplification (RCA) with φ29 DNA polymerase (Illustra TempliPhi Amplification Kit, GE Healthcare) as per the manufacturer’s instructions. The concatemeric DNAs were used directly for biolistic inoculation.

**Fig. 1:**
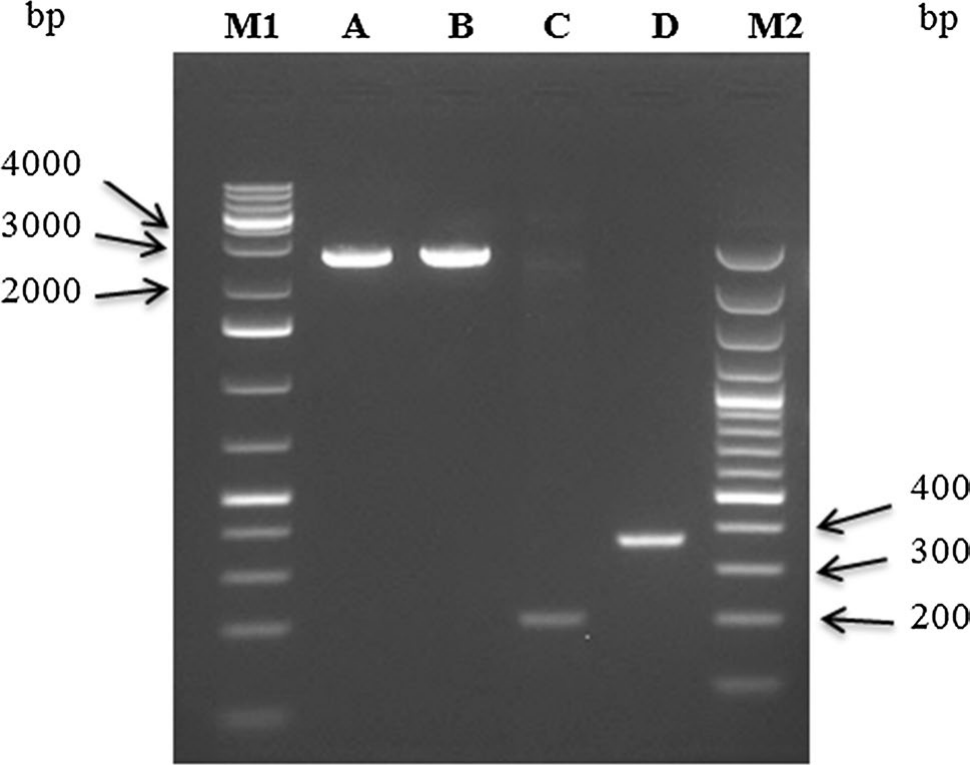
Amplification of DMV genomic components from naturally infected *Deinbollia borbonica*. Full-length DNA-A (1), full-length DNA-B, (2), CRA (3), and CRB (4). The DNA rulers used are 1 kb+ ladder (M1), and 100 bp ladder (M2)

The full-length DNA-A (GenBank accession no. KT878824) and DNA-B (GenBank accession no. KT878825) (Fig. 1A and B) were amplified from total DNA using Phusion High-Fidelity DNA Polymerase (Thermo Fisher Scientific) and partially overlapping abutting primers DNA-A_F_ (5′-ATAGGATCCTTTAGTTAATGAGTTTCCTGAC-3′) and DNA-A_R_ (5′- ATAGGATCCCACATATTGCTACGCGTC-3′) containing a natural BamHI restriction site (underlined), and primers DNA-B_F_ (5′-CCCTCTAGAGAGAGAAGCT-3′) and DNA-B_R_ (5′-CCCTCTAGAATCCTCATCGTCG-3′), containing a natural XbaI site (underlined). Apparent full-length PCR products were cloned into the pUC18 vector (Fermentas, USA), resulting in the plasmids pDMVA-1.0 and pDMVB-1.0.

**Fig. 2:**
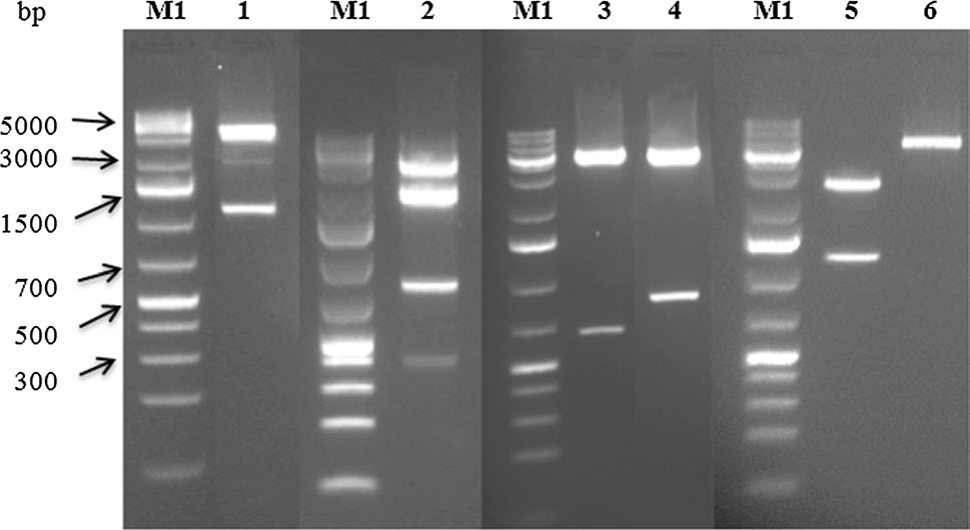
Restriction digestion of DMV DNA partial dimers used for biolistic inoculation. Selected restriction enzymes were used to verify the orientation of PCR fragments of DMV cloned into pUC18: BamHI/EcoRI-pDMVA-1.4 (1), BgIII-pDMVA-1.4 (2), EcoRV-pDMVA-1.4 (3), EcoRV-pDMVB-1.08 (4), BamHI/EcoRI-pDMVB-1.08 (5), and KpnI-pDMVB-1.08 (6). The DNA ruler used is 1 kb+ ladder (M1)

The Common Region (CR) of DNA-A (CRA) and DNA-B (CRB) was amplified with CRA and CRB primer pairs designed containing natural EcoRI, BamHI, and XbaI restriction sites (Table 1; Fig. 1C and D). To construct a partial repeat of DNA-A, a 0.4-mer (1,111 bp) EcoRI/BamHI fragment of CRA was inserted into the unique EcoRI and BamHI restriction sites of pUC18, and the resulting clone was designated pDMVA-0.4. The full-length BamHI/BamHI DMV-A fragment (2,852 bp) was digested from pDMVA-1.0 and inserted into the unique BamHI restriction site of pDMVA-0.4. The resulting clone containing 1.4 copies of DMV DNA-A was designated as pDMVA-1.4. A tandemly arranged partial repeat of DNA-B was constructed by inserting a 0.08-mer (209 bp) KpnI/XbaI fragment of CRB into the unique KpnI and XbaI restriction sites of pUC18, generating pDMVB-0.08. Insertion of the full-length XbaI/XbaI DNA-B fragment (2,702 bp) from pDMVB-1.0 into the unique XbaI restriction site of pDMVB-0.08 resulted in a clone harbouring 1.08 copies of DMV DNA-B and was designated pDMVB-1.08. The recombinant plasmids were introduced into *Escherichia coli* (DH5α) using the heat-shock transformation protocol. The orientation of the inserts was confirmed by restriction digestion (Fig. 2).

**Table 1:** Primers used for Deinbollia mosaic virus (DMV) amplification

Primer	Sequence (5´ → 3´)	T_a_ (°C)	Reference
CRA_R_^1,a^	ATAGGATCCCACATATTGCTACGCGTC	60	This study
CRA_F_^1,b^	CCCCCATGAATTCTTTAAAATGCTTTAG	60	This study
CRB_F_^2,c^	CCCTCTAGAGAGAGAAGCT	60	This study
CRB_R_^2,d^	TGGTACCAATAGCCTCCAAAAGCACGCA	60	This study
B4/F^2^	TTGGCTCTCAGGTGTCCACGT	63	[13]
B4/R^2^	ACGACCTCCATTACCTTCAACA	63	[13]

^1^ Primer pair used for the PCR amplification of DNA-A fragments

^2^ Primer pair used for the PCR amplification of DNA-B fragments

^a^ GGATCC = BamHI restriction site

^b^ GAATTC = EcoRI restriction site

^c^ TCTAGA = XbaI restriction site

^d^ GGTACC = KpnI restriction site

### Inoculation of plants by sap and biolistic inoculation

*Nicotiana benthamiana* was grown from seed, while cassava (*Manihot esculenta* Crantz) genotypes KME 1 and Mucericeri (which are highly susceptible to cassava mosaic disease [CMD]) were grown from virus-free stem cuttings obtained from Jomo Kenyatta University of Agriculture and Technology (JKUAT). To test the infectivity of DMV, virus-free test plants at the second- or third-true-leaf stage were inoculated by mechanically rubbing with homogenized plant sap and by biolistic delivery, with each experiment replicated three times.

Mechanical sap inoculation was carried out using inoculum derived from plants infected using concatemeric DNAs. Approximately 1 g of leaf tissue was ground with 0.2 mg of carborundum (300 mesh) in 500 μL of 0.2 M sodium phosphate buffer (pH 7.5) and used to inoculate the second set of true leaves of 10 plants of each test plant species. The inoculated plants were observed for symptom development until 35 dpi.

Concatemeric DNAs and DNA partial dimers were biolistically inoculated onto the meristem tissue using a hand-held particle acceleration device (Microdrop Sprayer II, Ascefran LLC, Raleigh, USA) at a pressure of 60 pounds per square inch (psi). For each experimental replicate, 10 test plants per plant species were inoculated with viral DNA, while two plants (negative controls) were inoculated with tungsten microprojectiles in water. Five µg of concatemeric DNAs or 2.5 µg of partial dimeric DNA clones of each genomic component was mixed with 50 µL of prepared 1.0-μm tungsten particles (BioRad, Hercules, CA). Aliquots of 10 μL per plant were used for bombardment in each experiment. Inoculated plants were kept in an environmentally controlled, insect-free growth chamber (25°C, 65% humidity and 16/8 h photoperiod) and monitored periodically for symptom development, beginning 14 days post-inoculation (dpi) and monitored daily for approximately 35 days. The progeny virus from the bombarded plants showing symptoms was inoculated back to *N. benthamiana* biolistically to satisfy Koch’s postulates for begomoviruses.

### Viral DNA detection by PCR amplification

To confirm infection in test plants between 14 and 21 dpi, apical leaves from inoculated plants were harvested for virus testing by PCR analysis. The DMV DNA-A common region (CRA) was amplified using the primer pair CRA/F (5’-TGTTGACAGGTGTTTGTTTTGC-3’) and CRA/R (5’-ATCACGAATTAGATCATGGCCC-3’). PCR amplification was performed in a total volume of 20 μL containing AccuPower PCR PreMix with dye (Bioneer, Korea), 0.1 μM each primer, and 40 ng of template DNA. Amplification was performed in a GeneAmp PCR System 9700 thermocycler (Applied Biosystems, Foster City, CA) using the following PCR program: an initial denaturation step at 94°C for 3 min; followed by 35 cycles of denaturation at 94°C for 30 s, annealing at 60°C for 1 min, and extension at 72°C for 1 min; and then a final extension step at 72°C for 10 min. The PCR products were analyzed in a 2% agarose gel stained with 0.025X GelRed (Biotium, USA).

### DNA probe labeling, hybridization and colorimetric detection

A non-radioactive, digoxigenin (DIG)-labeled probe was synthesized by PCR amplification using the primer pair B4/F and B4/R (Table 1), flanking the common region and nuclear shuttle protein gene (NSP), and pDMVB-1.0 according to the protocol of the digoxigenin (DIG) DNA Labeling and Detection kit (Roche Diagnostics, Indiana, USA). The reaction consisted of a denaturation step of 3 min at 94°C, 30 cycles of 30 s at 94°C, 1 min at 63°C and 1 min at 72°C, and a final extension step of 7 min at 72°C. The PCR products were analyzed in a 1.8% agarose gel. The probe was denatured at 95°C for 10 min and snap-chilled in cold water for 5 min to prevent re-annealing.

Total DNA (10 μL) for dot blot hybridization was obtained from each sample, denatured by heating for 5 min at 100°C in a water bath, and snap-chilled in ice water before blotted onto the nitrocellulose membrane (Hybond-N+, Amersham, UK). In tissue blot hybridization, fine cross-sections of leaf petioles were hand cut with a surgical blade and gently pressed with a roller onto the nitrocellulose membrane. Then, the membrane was air-dried, and the DNA was cross-linked to the membrane through exposure to ultraviolet light for 1 min in a Stratalinker UV Crosslinker (Stratagene, USA) at 120,000 microjoules. Pre-hybridization and hybridization steps were carried out at 42°C in a hybridization oven (Amersham Biosciences, UK) in DIG Easy Hyb Granules (Roche Diagnostics, Indiana, USA) as per the manufacturer’s protocol. The subsequent step of detection was done following the protocol of the DIG Nucleic Acid Detection Kit (Roche Diagnostics, Indiana, USA). Colorimetric-based detection was done using 5-bromo-4-chloro-3-indolyl phosphate (BCIP) and nitroblue tetrazolium salt (NBT), which produced a purple color on regions on the nitrocellulose membrane containing viral DNA.

## Results

### Infectivity and detection of DMV in *N. benthamiana* test plants

**Fig. 3:**
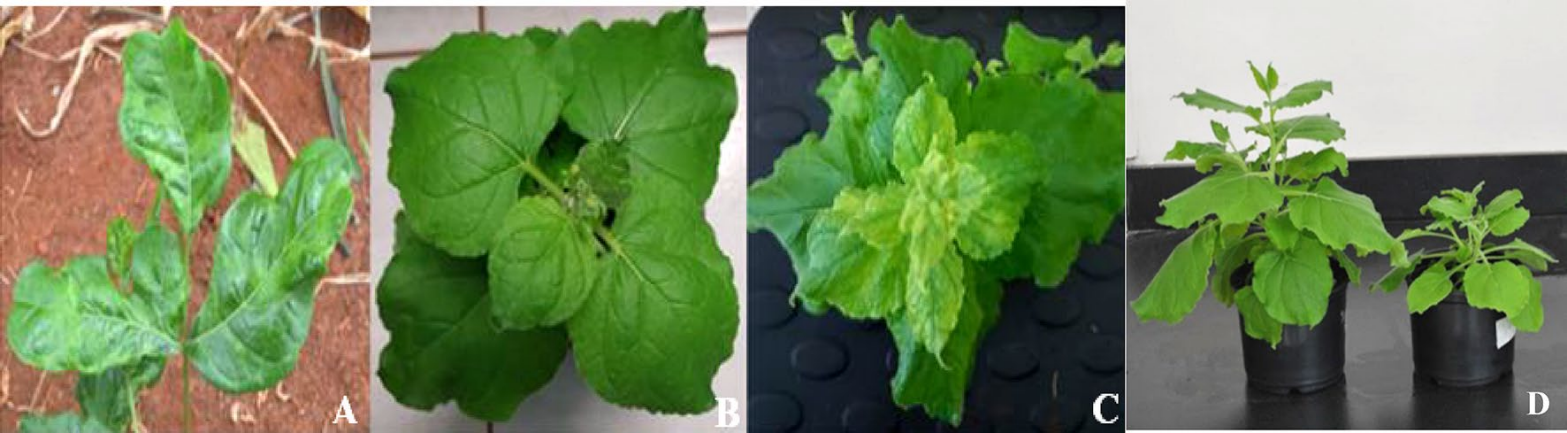
Symptoms exhibited by *D. borbonica* and *N. benthamiana* plants infected with DMV. Field-collected *D*. *borbonica* showing yellow mosaic symptoms (A), mock-inoculated *N*. *benthamiana* (B), *N*. *benthamiana* inoculated with DMV concatemeric DNAs exhibiting yellow mosaic symptoms at 21 dpi (C), *N*. *benthamiana* infected with DMV concatemeric DNAs showing stunting at approximately 28 dpi (D)

Sap and biolistically inoculated *N. benthamiana* with DNA partial dimers of either DNA-A or DNA-B failed to establish infection with DMV. On the other hand, biolistic inoculation of a combination of DNA partial dimers of DNA-A and DNA-B onto *N. benthamiana* caused 100% infection (30/30), with test plants exhibiting two types of symptom phenotypes: symptomatic infection (using concatemeric DNAs) and asymptomatic infection (using DNA partial dimers). The first symptom of infection appeared 21 dpi as yellow mosaic on the newly emerged leaves. At approximately 28 dpi, symptomatic plants exhibited some leaf yellowing and stunted growth with reduced leaf size relative to uninfected plants (Fig. 3). Some infected plants (50%, 15/30 plants) displayed a recovery phenotype, where symptom remission was observed but viral DNA was detectable by PCR, and dot blot analysis in young emerging leaves. All plants inoculated with DNA partial dimers were asymptomatically infected even after 35 dpi.

**Fig. 4:**
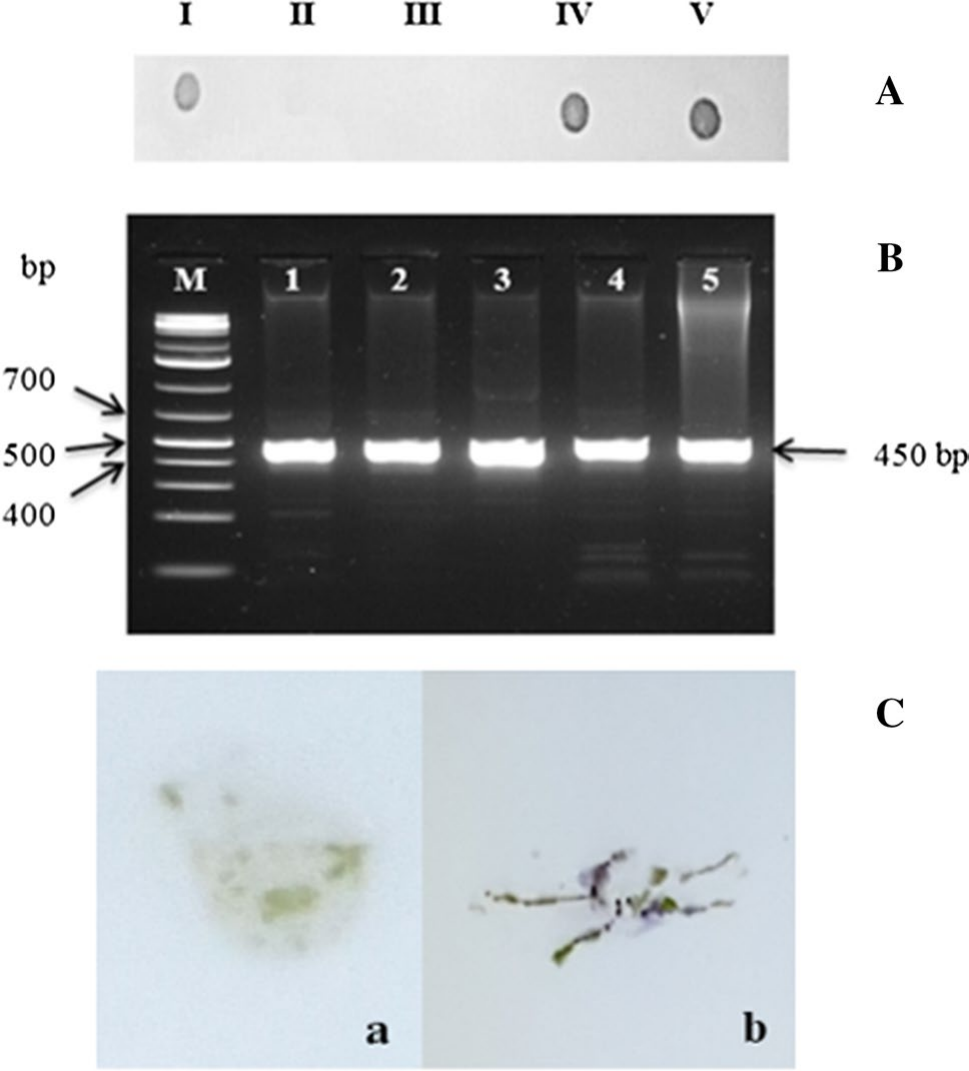
Detection of DMV by hybridization and PCR amplification. The presence of DMV was confirmed by the presence of a deep purple signal on the nitrocellulose membrane in dot and tissue blot hybridization assays and by the amplification of a 450-bp PCR product using the B4/F and B4/R primer pair. The detection signals shown are from a *D. borbonica* infected with wild-type virus (A I, and B 1), healthy *D. borbonica* (A II), mock-inoculated *N. benthamiana* (A III, and C a), *N. benthamiana* inoculated with DNA partial dimers (A IV, B 2, and B 3), and *N. benthamiana* inoculated with concatemeric DNAs (A V, B 4, B 5, and C b)

DMV was detected in infected *N. benthamiana* plants as a purple signal, while uninfected plants did not produce any signal in dot and tissue blot hybridization assays (Fig. 4A and C). PCR amplification of DNA-B from newly emerged uninoculated apical leaves of infected *N. benthamiana* plants using primer pair B4 (Table 1) resulted in a 450-bp amplicon (Fig. 4B), confirming the presence of the virus. PCR amplification and Sanger sequencing confirmed the presence of DNA-A and DNA-B in newly emerged uninoculated apical leaves of infected test plants using overlapping PCR primers as described by Kyallo et al. [13]. Viral DNA was not detected in mock-inoculated test plant controls.

### Infectivity of DMV in cassava test plants

**Fig. 5:**
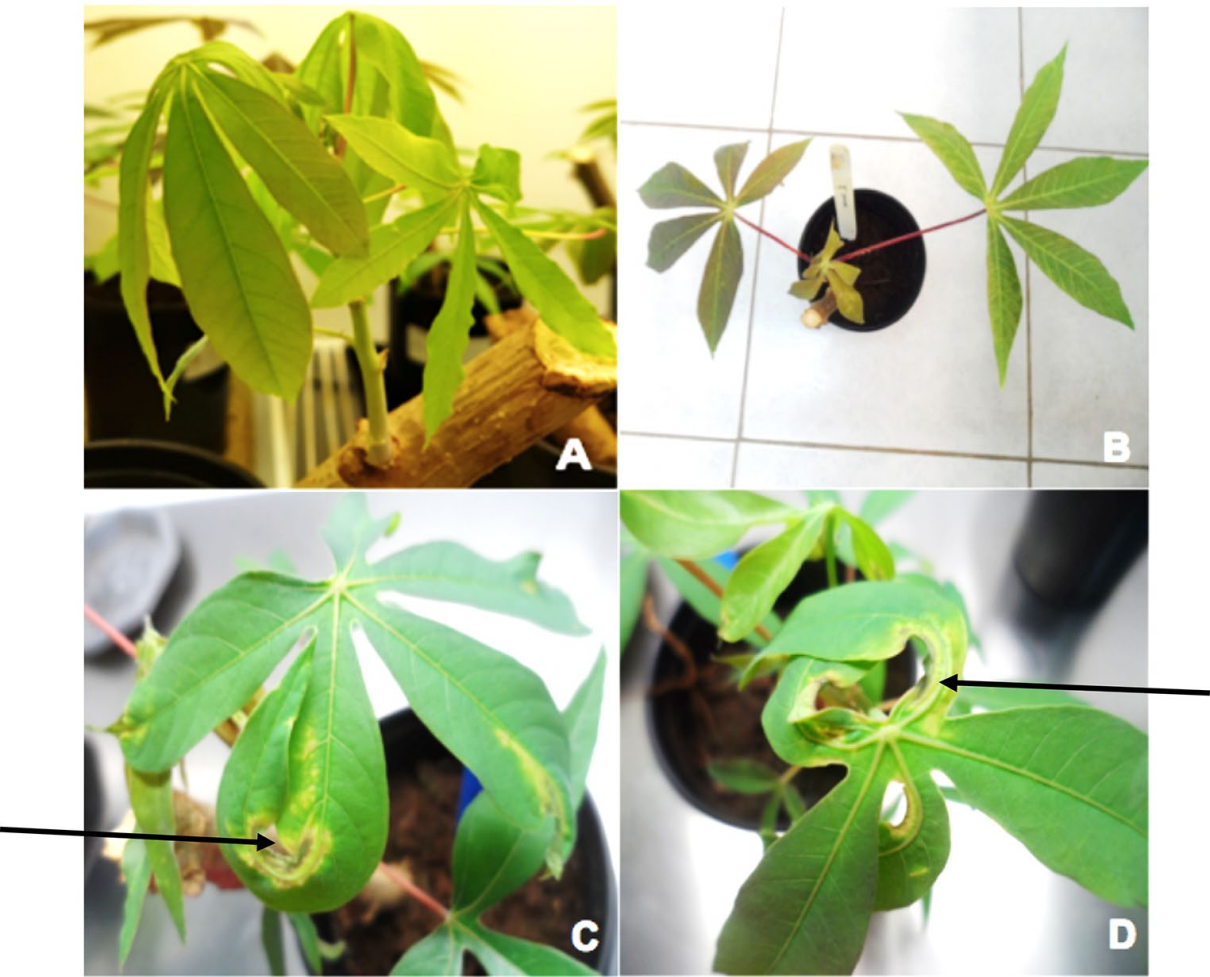
Hypersensitive response (HR) induced by DMV in cassava test plants. Cassava plants inoculated with concatemeric DNAs exhibited a hypersensitive reaction to the virus on leaves developing immediately after the point of inoculation. The photographs represent mock-inoculated test plant controls at approximately 14 dpi (A), and 28 dpi (B). Virus-inoculated plants exhibited spreading of necrosis along the main vein (shown by arrows) and misshaped leaflets 28 dpi (C, and D)

None of the biolistically inoculated cassava test plants developed symptoms in any of the three replicated experiments using sap from infected *N. benthamiana* plants, concatemeric DNAs, and DNA partial dimers. The mock-inoculated test plant controls did not exhibit any signs of infection or demonstrate any hypersensitivity reaction (HR) in the infectivity assay (Fig. 5A and B). However, necrosis was observed only on leaves that developed immediately after the point of inoculation on the meristem of virus-inoculated plants, and the leaf margins become misshaped and deformed (Fig. 5C and D). Viral DNA-A and DNA-B was not detected in the newly emerged leaves by PCR amplification and hybridization analysis. No symptoms were observed up to 35 dpi.

## Discussion

Biolistic inoculation to determine the infectivity of DMV was successful in *N. benthamiana*. Using concatemeric DNAs, systemic symptoms similar to those observed on infected *D. borbonica* in the field exhibiting yellow mosaic symptoms were induced. Also, the failure to cause infection using the sap inoculation method suggested that DMV was probably phloem-limited in infected plants.

Despite several attempts, we were unsuccessful in inducing infection in cassava and *N. benthamiana* using DNA partial dimers of single genomic components.The asymptomatic systemic infection exhibited by *N. benthamiana* plants inoculated with DNA partial dimers suggested that more-complex interactions existed between the natural host and the virus. Although we were unable to fulfill Koch’s postulates with the cloned components of DMV, we were able to demonstrate that DMV concatemeric DNAs were infectious after re-introduction through biolistics, facilitating the fulfillment of “Koch’s postulates” for begomoviruses. In the absence of seeds and after unsuccessful repeated transformation attempts, we were unable to use *D. borbonica* as a test plant in this study.

Geminiviruses trigger the gene silencing machinery of the host upon virus infection, which enables the infected host plants to recover [14]. Host recovery from geminivirus infection has been reported in several studies, with RNA silencing playing a direct role [15]. One such recovery pattern was seen in tomato plants which recovering from infection with tomato chlorotic mottle virus [16] and pepper plants recovering from infection with pepper golden mosaic virus [17]. Successful symptomatic infection of *N. benthamiana* by DMV is probably due in part to the hypersusceptible nature of this plant to virus infection, caused by a defective RNA-silencing-mediated host defense. [18, 19]. In test plants that exhibited the recovery phenotype, DMV was detectable even in uninoculated leaves [20]. Hypersensitive (HR) and necrotic responses are symptomatic manifestations of the plant’s host immune system triggered in virus-infected cells [21]. From our infectivity results, it may be concluded that cassava was not susceptible to DMV and that a resistant (or incompatible) host-virus interaction existed whereby HR was triggered upon virus infection and the resultant necrosis prevented subsequent establishment and movement of the virus.

Recombination, mutation and pseudo-recombination are responsible for driving the evolution of begomoviruses [22]. Globally, eight centers of diversification of geminiviruses exist, and East Africa is believed to be a center of diversity for begomoviruses [23]. So far, DMV has not been reported to be an economic threat, and thus is not an immediate concern for crop production, but it can be a potential threat and may cause problems in the future if it recombines with crop-infecting begomoviruses. There is potential for DMV to become pathogenic in a permissive host. The findings of this study suggest that there is resistance in cassava to DMV, which may be the case with many other viruses. However, high virus pressure and frequent occurrence of genomic recombination among begomoviruses may eventually result in resistance breakdown and lead to potentially serious problems to crops in the region. Recombination events have been directly implicated in the emergence of new begomovirus diseases and epidemics in cultivated hosts [24]. The devastating epidemics of cassava mosaic disease (CMD) caused by the Ugandan variant of East African cassava mosaic virus (EACMV-UG) in Uganda and neighboring countries [25], the tomato leaf curl virus (TYLCV) epidemics in the southern, central and northern parts of America, southern Asia, Africa and the Mediterranean basin [26], and the cotton leaf curl virus (CLCuV) epidemics in India and Pakistan [27] were all caused by a complex of begomoviruses including several recombinants.

One of the major obstacles to implementing effective disease surveillance and diagnosis is incomplete knowledge of the identity and level of genetic diversity occurring within populations of begomoviruses within a distinct region. Understanding the source(s) of new infections will facilitate the development of appropriate mitigation strategies against specific diseases in affected areas. It is certain that many undescribed begomoviruses are present in nature and are yet to emerge as a threat to crop cultivation in Africa. There is need for more studies to be done to identify other weed-infecting viruses that may present potential dangers to agriculture in the affected regions.
